# The coding region of the UFGT gene is a source of diagnostic SNP markers that allow single-locus DNA genotyping for the assessment of cultivar identity and ancestry in grapevine (*Vitis vinifera* L.)

**DOI:** 10.1186/1756-0500-6-502

**Published:** 2013-12-03

**Authors:** Silvia Nicolè, Gianni Barcaccia, David L Erickson, John W Kress, Margherita Lucchin

**Affiliations:** 1 Laboratory of Plant Genetics and Genomics, DAFNAE, University of Padova, Campus of Agripolis - Viale Università 16, 35020 Padova, Legnaro, Italy; 2 Department of Botany and Laboratories of Analytical Biology, National Museum of Natural History, Smithsonian Institution, P.O. Box 37012, Washington, DC 20013-7012 USA

**Keywords:** *Vitis vinifera* L., SNP-based genotypes, UFGT gene, Genetic identity of grapevine cultivars, Homonymy, Synonymy, Mislabeling

## Abstract

**Background:**

*Vitis vinifera* L. is one of society’s most important agricultural crops with a broad genetic variability. The difficulty in recognizing grapevine genotypes based on ampelographic traits and secondary metabolites prompted the development of molecular markers suitable for achieving variety genetic identification.

**Findings:**

Here, we propose a comparison between a multi-locus barcoding approach based on six chloroplast markers and a single-copy nuclear gene sequencing method using five coding regions combined with a character-based system with the aim of reconstructing cultivar-specific haplotypes and genotypes to be exploited for the molecular characterization of 157 *V. vinifera* accessions. The analysis of the chloroplast target regions proved the inadequacy of the DNA barcoding approach at the subspecies level, and hence further DNA genotyping analyses were targeted on the sequences of five nuclear single-copy genes amplified across all of the accessions. The sequencing of the coding region of the UFGT nuclear gene (UDP-glucose: flavonoid 3-0-glucosyltransferase, the key enzyme for the accumulation of anthocyanins in berry skins) enabled the discovery of discriminant SNPs (1/34 bp) and the reconstruction of 130 *V. vinifera* distinct genotypes. Most of the genotypes proved to be cultivar-specific, and only few genotypes were shared by more, although strictly related, cultivars.

**Conclusion:**

On the whole, this technique was successful for inferring SNP-based genotypes of grapevine accessions suitable for assessing the genetic identity and ancestry of international cultivars and also useful for corroborating some hypotheses regarding the origin of local varieties, suggesting several issues of misidentification (synonymy/homonymy).

## Findings

### Introduction

In the Vitaceae family, the genus *Vitis* is of great agronomic importance in temperate areas. Within this genus, the only European species, *Vitis vinifera* L., represents one of the oldest cultivated plants and is the only species extensively used in the global wine agro-industry [1]. The vast majority of the world’s grapes are produced by cultivars of the diploid *V. vinifera* subsp. *vinifera* (2*n* = 2*x* = 38), and nearly all cultivars are highly heterozygous, hermaphroditic and cleistogamous, although they out-cross easily [2]. Cultivated grapevine is derived from the wild ancestor *V. vinifera* subsp. *sylvestris* that underwent several drastic morphological and physiological changes during domestication, such as in reproductive behavior (*i.e.,* from out-crossing to selfing), berry and bunch size, seed and flower morphology, higher sugar content, and greater and more regular yields [3]. New genotypes are produced by sexual reproduction, and then the diffusion of cultivars with desirable traits is fulfilled through the vegetative propagation of cuttings. The marked heterozygosity of grapevine genotypes, the need to dispose of cultivars with stable morphological traits and the high incidence of inbreeding depression have forced wine growers to adopt asexual propagation to ensure the maintenance of plantation features [4]. Although clonal multiplication should ensure genetic homogeneity, the occurrence of somatic mutations may eventually lead to the formation of clonal variants and genetic chimerisms, when one or more genetic mutations take place in only one cell layer of the plant [5]. Because of these large sources of genetic variability, the frequent introduction of plant material into numerous secondary centers of domestication and the eventual hybridization between the domesticated forms and the wild ancestors, thousands of grapevine cultivars and even biotypes within cultivars exist and are generally classified according to their final product, wine, table grapes or raisins [6,7]. Because of the occurrence of several cases of synonymy and homonymy among the grapevine genotypes, the number of grapevine cultivars available in worldwide germplasm collections is estimated to be around 10,000-14,000 according to different authors [3,8], but their exact origin is still uncertain. Italy likely represents one of the richest countries in ampelo-biodiversity, counting around 2,000 cultivars compared to only 400 present in France, due to both native grapevines, not wholly officially registered, and the massive presence of regional minor vineyards [9]. Despite this large biodiversity richness, only a small number of grapevine cultivars are employed for global wine production, which contributes to the genetic erosion and loss of variability in all those countries where viticulture practice is very common, such as in Italy, Spain and France [10]. Consequently, the identification and characterization of grapevine cultivars is necessary and must be ensured both for resolving frequent miscalling events and for preserving ancient local germplasm accessions that represent an irreplaceable resource of genes and genotypes that are potentially useful for breeding programs.

Properly recognizing grapevine cultivars is complex to achieve and, because of the high adaptability and plasticity of the species *V. vinifera* to different environmental conditions, misidentification is common. Accurate characterization of grapevine germplasm relies on the choice of appropriate investigative tools. In addition to the traditional ampelography and ampelometry methods strongly influenced by plant phenology, alternative approaches based on molecular markers have been developed to guarantee the identification of both grapevines and, when possible, vine-derived products, such as juice and wine, to which morphological assays are clearly not applicable [11]. Among the principal molecular markers exploited, simple sequence repeats (SSR) markers represent one of the most suitable diagnostic tools currently adopted by the international scientific community to define a cultivar and to reconstruct its genealogy. After This et al. [12], a set of six SSR loci based on di-nucleotide repeats were chosen as an appropriate marker system for the genetic characterization and identification of cultivars (http://www.vivc.de). Recently, an additional set of microsatellites with longer core repeats were isolated and proposed to implement grapevine genotyping to avoid common problems of allele calling [13]. Another class of discriminant markers is represented by single nucleotide polymorphisms (SNPs), single base-pair differences in the form of substitutions or insertions/deletions (In/Dels), which are sources of huge genetic variation in the grapevine genome [14]. These DNA markers are widely used in animal and human genome analysis, whereas few works have exploited them for identification purposes in the major crop plants [15]. The employment of SNP markers could expedite the automation and precision of characterization procedures because the sequence information of a nucleotide snippet could be sufficient for genotyping grapevine cultivars, also allowing an actual standardization among laboratories [16]. Recently, a set of 48 SNP variants proved to represent a very robust genetic identification system, highly stable and repeatable, and with a discriminating power comparable to a set of 15 SSR markers [17]. In addition, SNP markers showed a very low rate of genotyping errors and a low appearance of new mutations when compared to SSR markers, avoiding any allele binning and allowing for prompt databasing and direct comparison of data arising from different laboratories [17]. The availability of the complete sequence of the grapevine nuclear genome encouraged the analysis of allelic diversity and SNP discovery in genes that also control important traits [18,19].

DNA barcoding is a technique for characterizing species of organisms using a short DNA sequence from a standard and agreed-upon position in the genome. This standardized region is then compared to a public reference library of species identifiers in order to assign unknown specimens to known species (http://www.barcodeoflife.org/). In a broader sense, DNA barcoding is a genomic approach based on the detection of SNPs from one or few target loci used to identify an unknown organism by matching DNA sequence recovered from the sample to a database of sequences from known organisms that have been previously described and recognized using morphological keys [20]. The methodology applied at the species level lies in the analysis of the mitochondrial and chloroplast genome to recognize, respectively, animal or plant organisms. The employment of DNA barcoding at the sub-species level, instead, is not a conventional application of the methodology. Consequently, this research aims to assess the applicability of chloroplast DNA barcoding to unambiguously distinguish varietal genotypes of *V. vinifera*[21]. Since the genetic distance among subgroups within a species is generally too small to allow the definition of a genetic threshold to delimitate different varieties, a character-state DNA sequencing procedure based on single-copy nuclear genes was also developed [22]. This technique could be of great utility for the correlation of genetic diversity with phenotypic variability and, hence, for the definition of cultivar-specific genotypes that are exploitable for authentication assays.

The final goal of this study is to implement genomic approaches useful to distinguish grapevine subspecies entities to both safeguard the germplasm patrimony of the species, for instance protecting local varieties and resolving cases of homonymy and synonymy, and warrant the authenticity of the grapevine cultivars and their derivatives.

### Materials and methods

#### Germplasm sampling of Vitis spp

For the molecular analysis, we sampled leaves from 164 accessions of *Vitis* spp*.*, including a large collection of cultivars of *V. vinifera* having different origin, diffusion and utilization, two interspecific hybrids (Bianca and the local cultivar Tintoria) and five wild species (*V. riparia*, *V. rupestris*, *V. berlandieri*, *V. cinerea* and *V. labrusca*) used as out-groups (see Additional file 1). Of the 157 cultivars of *V. vinifera*, representative of different genotypes, belonging to international, national or local accessions, selected among the most common cultivars throughout Europe destined for wine production, table grapes and raisins, we employed 135 international certified cultivars, including one accession named Perla present in our collection of the University of Padua, and 22 local cultivars widespread in the Venetian region. In detail, the 134 international certified *V. vinifera* accessions, mainly from Europe (*i.e*., 54 from Italy, 22 from Spain, 19 from France, 15 from Portugal, 1 from Rumania, 9 from Greece, 3 from Moldova, 3 from Turkey, 2 from Croatia, 1 from UK, 1 from Siria, 1 from Germany, 1 from Austria, 1 from Balkan area and 1 from USA), were supplied by certified commercial nurseries, whereas the putative *V. vinifera* accession Perla was obtained from Hungary. Regarding the ancient local cultivars, one hybrid (Tintoria) and 22 accessions of *V. vinifera*, originating from Northeastern Italy, in particular from Breganze (Vicenza) and from Euganea Hills (Padova), and maintained in the experimental farm of the University of Padova, were analyzed as particular case studies.

#### Genomic DNA extraction

Total genomic DNA was isolated from frozen young leaf tissues using the DNeasy extraction kit (Qiagen) according to the manufacturer’s protocol. Each DNA sample was eluted in 80 to 100 μl of 0.1× TE buffer (Tris–HCl 100 mM, EDTA 0.1 mM pH = 8), and the purity, integrity and quantity of all DNA samples were estimated by electrophoresis on a 0.8% agarose/1× TAE gel by comparison with a 1 Kb Plus DNA ladder (Invitrogen) of known concentration.

### DNA barcode markers, single-copy nuclear gene markers and PCR assays

The barcoding approach was carried out by amplifying and sequencing six chloroplast markers, including the *rps16* intron and the *trnH-psbA*, *rpl32*-*trnL*, *trnT-*trnL, *trnL-trnF* and *atpB-rbcL* intergenic spacers. Standard barcodes such as *rbcL* and *matK* were discarded *a priori* because of the well known modest discriminatory power in resolving different but closely related species of the former [23] and the multiple failed amplifications along with low sequence quality experienced using the latter [21,24,25].

The genotyping approach was based on three nuclear single-copy genes and two cDNA sequences (coded as ID04 and IIC08) belonging to a *V. vinifera* EST database containing sequences related to four functional classes of genes, such as sugar metabolism, cell signaling, anthocyanin metabolism and defense related [26]: the GAI gene, involved in the giberellic acid mediated signaling [27]; an ATP synthase gene [28]; and UFGT (UDP-glucose: flavonoid 3-0-glucosyltransferase) gene, the key enzyme for the accumulation of anthocyanins in berry skins [29]. For SNP genotyping purposes, among the nuclear markers it was essential choosing single-copy genes to avoid problems associated to the identification of orthologous genes in different grapevine accessions. In fact the existence of duplicated copies of candidate genes would have implied the presence of multiple alleles creating difficulties in the attribution of the origin to the sequence variants [28,30]. Some genes, such as GAI and ATP synthase, were selected because previously investigated in phylogenetic analysis within the Vitaceae family, showing to be highly informative in terms of discriminant polymorphisms [27,28]. Additionally, we also selected two EST sequences and a portion of the UFGT gene that proved to be similarly efficient for assessing genetic diversity in grapevine [26].

For each chloroplast and nuclear marker, the PCR reactions were conducted in a volume of 25 μl containing 15 ng of genomic DNA as template, 1× PCR buffer (100 mM Tris–HCl pH 9.0, 15 mM MgCl2 and 500 mM KCl), 0.2 mM dNTPs, 0.2 μM of each primer and 0.5 U of *Taq* DNA polymerase. The primers pairs, along with the relative nucleotide sequences and the corresponding references, are supplied in Table 1. All PCR amplifications were performed on a GeneAmp PCR System 9700 (Applied Biosystems). The thermocycling conditions for the chloroplast regions were the following: 5 min at 95°C followed by 35 cycles of 30 sec at 95°C, 1:10 min at 55°C to 63°C (in function of the marker) and 1:20 min at 72°C, followed in turn by 7 min at 72°C and then held at 4°C. Positive and negative controls were used as reference standards. The PCR-derived fragments were resolved on 2% agarose/TAE gels and visualized under UV light using Sybr Safe staining. All amplification products were purified by gel filtration with Sephadex G-50 (Amersham Pharmacia Biotech) and then directly sequenced bidirectionally on an ABI3100 automated sequencer (Applied Biosystems).

**Table 1:** List of primers used for each chloroplast and nuclear marker with their chromosome localization, function, amplicon length, primer nucleotide sequences and references

**Marker**	**Localization**	**Coding region**	**Length (bp)**	**Primer name**	**Primer sequence (5′-3′)**	**Ta (°C)**	**References**
*rps16*	Chloroplast	Intron	956	rps_F	GTGGTAGAAAGCAACGTGCGACTT	56	[31]
				rps_R	TGCGGATTCCTAAGAGCAGCGT		[31]
*rp132-tmL*	Chloroplast	Intergenic spacer	1377	trnLUAGR	CTGCTTCCTAAGAGCAGCGT	50	[32]
				rpl32_F	CAGTTCCAAAAAAACGTACTTC		[32]
*trnH-psbA*	Chloroplast	Intergenic spacer	460	psbA3′f	GTTATGCATGAACGTAATGCTC	56	[33]
				trnHf	CGCATGGTGGATTCACAATCC		[34]
*trnT-tmL*	Chloroplast	Intergenic spacer	1016	trnTUGU2F	CAAATGCGATGCTCTAACCT	56	[35]
				5′tmLUAAR	TCTACCGATTTCGCCATATC		[36]
*atpB-rbcL*	Chloroplast	Intergenic spacer	927	atpB-rbcL_F	AACACCAGCTTTRAATCCAA	56	[37]
				atpB-rbcL_R	ACATCKARKTACKGGACCAATAA		[37]
*trnL-trnF*	Chloroplast	Intergenic spacer	406	trnL_UNIE	GGTTCAAGTCCCTCTATCCC	50	[36]
				trnL_UNIF	ATTTGAACTGGTGACACGAG		[36]
GAI	Chromosome 1	Transcription factor for GA	761	GAI_F	ATGGATGAGCTTCTGCTGT	50	[27]
				GAI_R	TAGAAGTGCATCCTGRAGAAT		[27]
ID04	Chromosome 3	ZIP DNA-binding protein	419	Id04_F	CACCAGTCCCTTACCAGTCT	55	[38]
				Id04_R	CAGTAGAGGAACACAACTGAG		[38]
IIC08	Chromosome 3	ZINC finger protein	418	IIC08_F	CAAGGCCTTCTCTTCGTACC	60	[38]
				IIC08_R	AAGAATTCATATCGCCGACC		[38]
ATP	Chromosome 7	ATP synthase	800	ATP_F	ATGCTGTTCCAGTCCGTTTC	60	-
				ATP_R	GGGTCGATGGTGATCTTCT		-
UFGT	Chromosome 16	Glucosyltransferase	919	UFGT_F4	ATGTCTCAAACCACCACCAACC	63	-
				UFGT_R3	TGACGGTGCCAAAGCTAATG		-

#### Analysis of marker data

All of the obtained chromatogram files were visualized and manually edited by means of Sequencer 4.8 (Gene Codes Corporation, Ann Arbor, MI, USA). Nucleotide sites in which only a single nucleotide (referred to as characteristic attribute, CA, according to [22]) was detected per site were considered homozygous, whereas when two CAs per site were found, the position was considered heterozygous and recorded using the IUB (International Union of Biochemistry) conventional code for degenerate bases. Sequence similarity searches were performed using the GenBank BLASTn algorithm (http://www.ncbi.nlm.nih.gov/BLAST) against the nucleotide databases of NCBI to check the correspondence between the sequences of the obtained amplicons with the expected sequences. Multiple sequence alignments for each marker alone and for the combined sequence derived by the five regions were performed by the software SeAl (version 2.1, University of Edinburgh, Scotland, UK).

Measures of genetic variation were used to estimate the levels of polymorphism within *V. vinifera* cultivars as well as among *V. vinifera* and *Vitis* outgroups. The inter- and intraspecific genetic divergences were carried out within and between different *V. vinifera* accessions according to the Kimura-2-Parameter distance model [39] using MEGA 4.1 beta software (The Biodesign Institute, Tempe, AZ, USA). Based on the pairwise nucleotide sequence divergences, the neighbor-joining (NJ) tree was estimated and rooted using the accessions from different species as outgroups. A bootstrap statistical analysis was conducted to measure the stability of the computed branches with 1,000 resampling replicates. In addition, descriptive genetic diversity and differentiation statistics were conducted over all marker loci for each geographical accession group to estimate the levels of polymorphism within and between different grapevine cultivars using the software POPGENE (version 1.21, University of Alberta, Edmonton, AB, Canada). In order to perform this analysis, eight large population groups within *V. vinifera* plus an outgroup of *Vitis* non-*vinifera* were delineated in the total sample. In some cases, the cultivars were reattributed to the population groups according to the main current geographical diffusion of the cultivation and the eight different regions identified were called: Local, Italy (including cultivars from Austria and Croatia), Central Europe (with cultivars from France, UK and Germany), Spain, Portugal, Eastern Europe (grouping cultivars from Hungary, Rumania and Moldavia), Near East (including cultivars from Siria and Turkey) and Balkan Peninsula (with cultivars from Greece and Balkan area). The observed number of alleles (no) and the effective number of alleles (ne) per locus were calculated according to Kimura and Crow [40]. The Shannon’s information index of phenotypic diversity (I), the Nei’s genetic diversity (H) and the Wright’s (1978) fixation index (Fis) were also computed to summarize the data of nuclear SNP markers in *V. vinifera*.

The population structure of our *V. vinifera* accessions was investigated using the model-based (Bayesian) clustering algorithm implemented in the software STRUCTURE version 2.2 (University of Chicago, IL, USA). This software was exploited to assign individual genotypes, predefined according to the nine geographical groups introduced previously, to clusters inferred according to marker allele combination and distribution. All simulations were carried out assuming an admixture model, with no *a priori* population information and with correlated allele frequencies. To evaluate the appropriate K value, the software was run ten independent times for each K value (from 1 to 10) using a burning period of 100,000 and 100,000 Markov chain Monte Carlo (MCMC) repeats. Estimation of the most likely value of K was done as recommended by Evanno et al. [41]. Accessions with membership coefficients of qi > 0.7 were assigned to a specific group, whereas accessions with qi < 0.7 were identified as admixed.

Because of the intrinsic difficulty in applying chloroplast DNA barcoding at the subspecies and population levels, a second approach combining the sequencing of nuclear genes with a character-based method was developed [42]. The information about SNP occurrence was adopted to define the genotyping matrix. In case of heterozygous sites, the genotype was defined without separating the two nucleotides found for each heterozygous polymorphic position and recording its state with the IUB code. The presence of specific character states and combination of character states was evaluated as distinctive of a particular cultivar or, more generally, of a group of cultivars within *V. vinifera*. The terms “pure”, “simple” and “compound” were employed in agreement with the terminology proposed by DeSalle et al. [22]: pure indicates a CA shared among all the individuals belonging to a genotype and absent from the others; simple describes a CA narrowed to a single nucleotide position; and compound refers to a combination of particular CAs at determined multiple nucleotide positions.

### Results

#### DNA barcoding of chloroplast sequences

In a number of preliminary assays, we targeted six different chloroplast markers for barcoding grapevine accessions: the *rps16* intron and the *trnH-psbA*, *rpl32*-*trnL*, *trnT-trnL*, *trnL-trnF* and *atpB-rbcL* intergenic spacers. Based on the available literature, these sequences were included in the most polymorphic regions widely used for genetic identity or molecular phylogeny studies of various plant taxa [25,32,37,43,44]. Differently to what reported for other crop plants (see [21] and references therein), the *trnH-psbA* intergenic spacer was found to be not only monomorphic among different *V. vinifera* cultivars, but also poorly polymorphic among *Vitis* species, scoring only two SNPs. Additional chloroplast regions were tested by analyzing only a core subset of 30 *V. vinifera* accessions, including also representative samples for each *Vitis* species. An unexpected lack of polymorphisms was observed both at the intraspecific and interspecific level (data not shown).

Because of the inadequacy of the chloroplast genome for DNA barcoding purposes, further analyses were targeted on the sequences of five nuclear single-copy genes amplified across all of the accessions.

#### Discovery and frequency of SNPs on single-copy nuclear genes

The universal primers designed on the novel nuclear gene targets proved to be highly effective in generating single and reliable amplicons, with an estimate of successful amplification equal to 100%.

Sequences for a total length of 3,317 bp were investigated at the nucleotide level over all nuclear DNA target regions for each accession and then used for the discovery of informative SNPs and analysis of their nature and frequency (no In/Dels were recovered). Because the occurrence of SNPs, in either homozygous or heterozygous states, had to be detected with a high degree of confidence to infer the genotype composition suitable for cultivar characterization aims, we focused our attention on the nucleotide positions with no cases of ambiguous base calling. When comparing all the genotypes, a total of 107 and 96 polymorphic sites were recorded among *Vitis* species and among *V. vinifera* accessions, respectively, with an average frequency of 1 SNP/31.00 nucleotides and 1 SNP/34.55 nucleotides, respectively (Table 2). Considering the single regions individually, the average frequency of CAs ranged from 1 SNP/19.95 nucleotides to 1 SNP/54.36 nucleotides for the regions ID04 and GAI, respectively (Table 2).

**Table 2:** **Basic information on the nuclear barcode regions with the number and the frequency of SNPs occurrence within *Vitis vinifera *** **and between *Vitis *** **spp. along with the haplotype number (Hn) and accessions numerosity (Nh) for each barcode region**

	**No. SNPs**		**Frequency (1SNP/bp)**		**Hn**		**Nh**
	***Vitis *** **spp.**	***V. vinifera***	***Vitis *** **spp.**	***V. vinifera***	***Vitis *** **spp.**	***V. vinifera***	***V. vinifera***
GAI	17	14	44.76	54.36	23	18	1-101
ID04	21	21	19.95	19.95	33	28	1-44
IIC08	19	17	22.00	24.54	14	11	2-86
ATP	21	15	38.09	53.33	25	19	1-73
UFGT	29*	29*	31.69	31.69	97	92	1-15
Combined	107	96	31.00	34.55	134	126	1-4

*only a selection of SNPs.

#### SNP-based genetic diversity descriptive statistics

**Figure 1:**
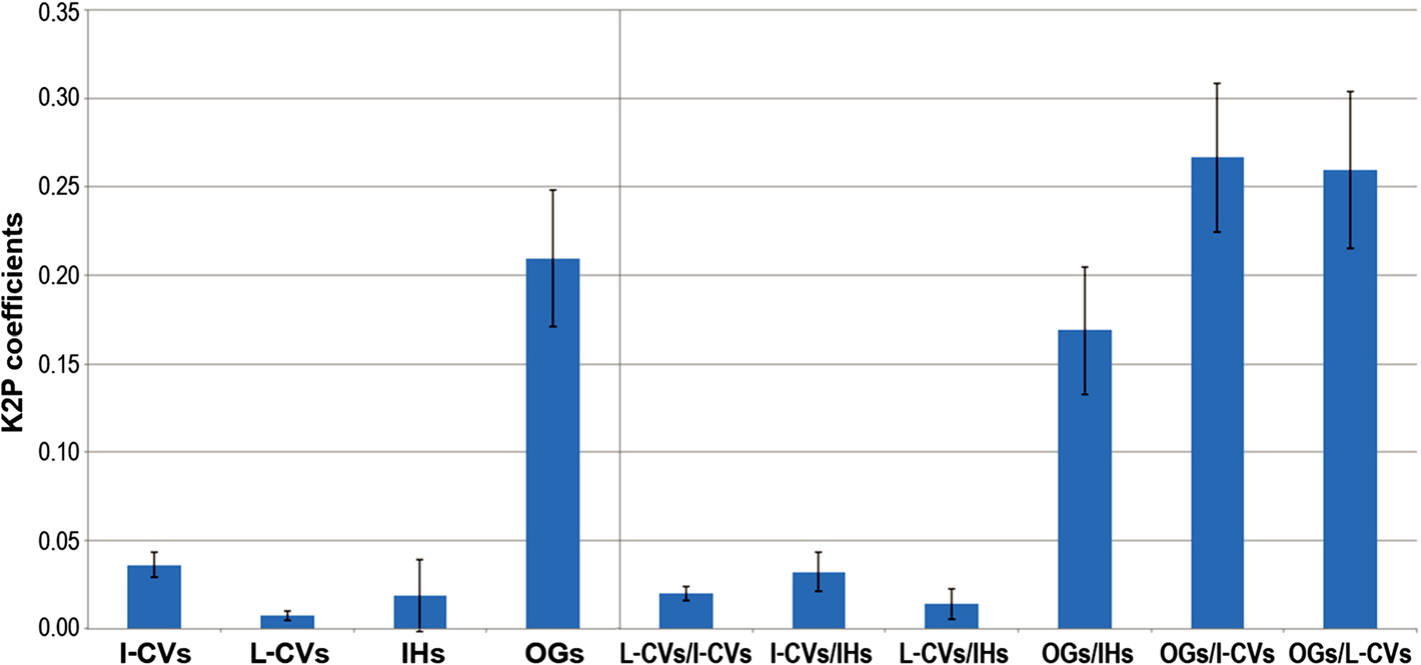
**Histograms of intraspecific and interspecific genetic distance estimates calculated in pair-wise comparisons of *Vitis *germplasm within and between accession groups using the Kimura 2-parameter on the basis of all barcode regions, along with bars for standard deviation values.** The subgroups include international cultivars (I-CVs) and local cultivars (L-CVs) of *V. vinifera*, outgroup accessions of wild *Vitis* species corresponding to *V. labrusca*, *V. cinerea*, *V. berlandieri*, *V. rupestris* and *V. riparia* (OGs) and interspecific hybrids (IHs).

Genetic diversity among *V. vinifera* accessions was investigated and, for this aim, the whole germplasm collection was split into four subgroups: i) the international cultivars; ii) the local cultivars, that will be analyzed in great detail; iii) the interspecific hybrids, Bianca that represents a *V. vinifera* backcross with introgressed genes from non-*V. vinifera* ancestors, and the local accession Tintoria; and iv) the five *Vitis* species used as outgroups. As reported in Figure 1, the highest genetic variation expressed in terms of K2P coefficients was scored for the outgroups (d = 0.21), whereas genetic variation estimates were very low within *V. vinifera* accessions. The genetic diversity, computed for each of the four subgroups, was equal to 0.04, 0.01, 0.02 and 0.21, respectively. The genetic distance estimates among subgroups were 0.02 between local and international cultivars, 0.03 and 0.01 between interspecific hybrids and international cultivars and local cultivars, respectively, and 0.27, 0.26 and 0.17 between outgroups and international cultivars, local cultivars and interspecific hybrids, respectively.

Classical descriptive statistics of the grapevine germplasm were estimated at single loci for individual subgroups and over all accession groups (Table 3). The observed and expected number of alleles per single subgroup of *V. vinifera* accessions varied from 1.22 to 1.63 and 1.18 to 1.29, respectively. The average estimate of the Shannon’s information index of phenotypic diversity for molecular profiles was I = 0.21 and I = 0.19 for certified and local cultivars, respectively, whereas the mean Nei’s genetic diversity, equivalent to the expected heterozygosity, was as low as H = 0.13 and H = 0.12 for certified and local cultivars, respectively (Table 3).

**Table 3:** Summary of genetic variation statistics for nuclear DNA markers, including the total number of alleles (S), the percent of polymorphic sites within the geographical sub-population, the observed (no) and expected (ne) number of alleles, the observed (Ho) and the expected (He) heterozygosity, along with the Shannon’s information index of phenotypic diversity and the total Nei’s expected heterozygosity (H) over all common grapevine accessions

**Accessions**	**Sample size**	**P (%)**	**na**	**ne**	**I**	**H**	**Fis**
Local	46	55 (50.46%)	1.53	1,19	0,19	0.12	−0.22
Portugal	30	41 (37.61%)	1.39	1.19	0.18	0.12	−0.13
Spain	44	45 (41.28%)	1.44	1.22	0.19	0.13	−0.13
Italy	114	54 (49.54%)	1.51	1.19	0.19	0.12	−0.01
Central Europe	42	46 (42.20%)	1.45	1.19	0.19	0.12	0.12
Eastern Europe	12	67 (61.47%)	1.63	1.29	0.28	0.18	0.10
Balkan Peninsula	20	42 (38.53%)	1.40	1.22	0.20	0.13	−0.11
Near East	8	24 (22.02%)	1.22	1.18	0.14	0.10	−0.33
Locals	46	55 (50.46%)	1.53	1.19	0.19	0.12	−0.22
Cultivars	270	89 (81.65%)	1.90	1.20	0.21	0.13	−0.05
Outgroups	10	67 (67.47%)	1.74	1.40	0.36	0.23	0.34
Total	326	108 (99.08%)	2.22	1.21	0.24	0.14	−0.04
St. Dev.	n.a	n.a	0.50	0.28	0.23	0.16	n.a

**Figure 2:**
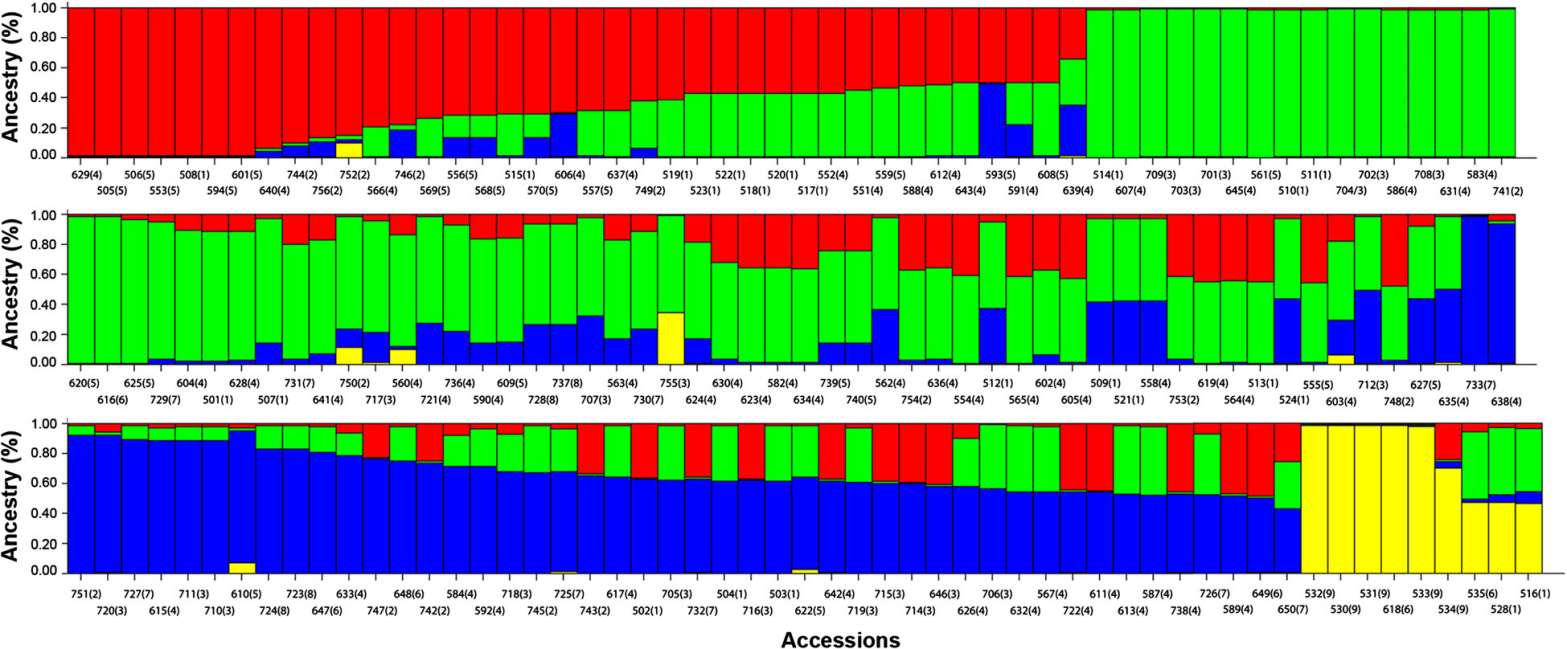
**Population structure of *Vitis *germplasm collection as estimated with STRUCTURE software.** Each accession is represented by a vertical histogram portioned into K = 4 colored segments that represent the estimated membership of each individual genotype. Accessions were ordered by inferred clusters and identified by a the accession number and a number between brackets that corresponds to the cultivation area, *i.e*. local cultivars (1), certified cultivars from Portugal (2), Spain (3), Italy (4), Central Europe (5), Eastern Europe (6), Balkan Peninsula (7), Near East (8) and wild *Vitis* species (9) (see also Additional file 2 for details on single subgroups and inferred clusters).

**Figure 3:**
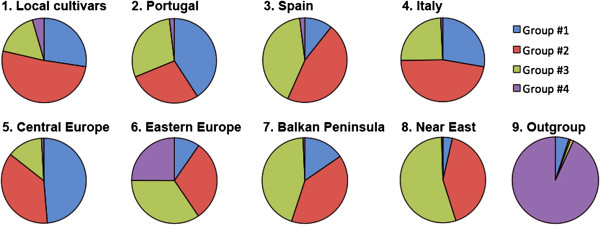
**Graphical distribution of the proportions of membership of analyzed *Vitis *accessions.** The accessions are organized in nine subgroups (*i.e*., local cultivars and certified cultivars of *V. vinifera* divided according to cultivation area, outgroups identified with non-*V. vinifera* species and interspecific hybrids) in each of the three inferred genetic clusters (see Additional file 2 for the specific values of membership).

Investigation of the population structure by the estimation of ΔK suggested that our germaplasm collection of accessions is most likely composed of four genetically distinguishable groups (K = 4), as shown in Figure 2. A low genetic homogeneity was observed within each subgroup, and most of the accessions revealed an admixed origin sharing large fractions of genetic background. In particular, it appears evident that there is not a strict relationship between the genetic composition of the inferred clusters and the geographical origin of cultivar recovery (Figure 3). In fact, apart the American outgroups that cluster separately in a specific subgroup along with Perla, all the remaining cultivars were assigned to the other three groups identified by the software independently by the cultivation area. Proportions of membership to the four inferred clusters for each geographical subgroup were also calculated (see Additional file 2). The most differentiated accessions corresponded to the non-*V. vinifera* species that scored 93% of individuals assigned to single genetic cluster, whereas the other accessions seemed to be more genetically related, sharing several nucleotides across all SNP sites and hence proving hybridization events between the gene pools. Some cultivars were found strictly related to non-*V. vinifera* species, including Perla, two interspecific hybrids, Tintoria and Bianca, and the old accession Gruaja. The cultivars from Eastern Europe showed the highest contribution by non-*V. vinifera* species.

#### Specific character-based genotypes of international cultivars

**Figure 4:**
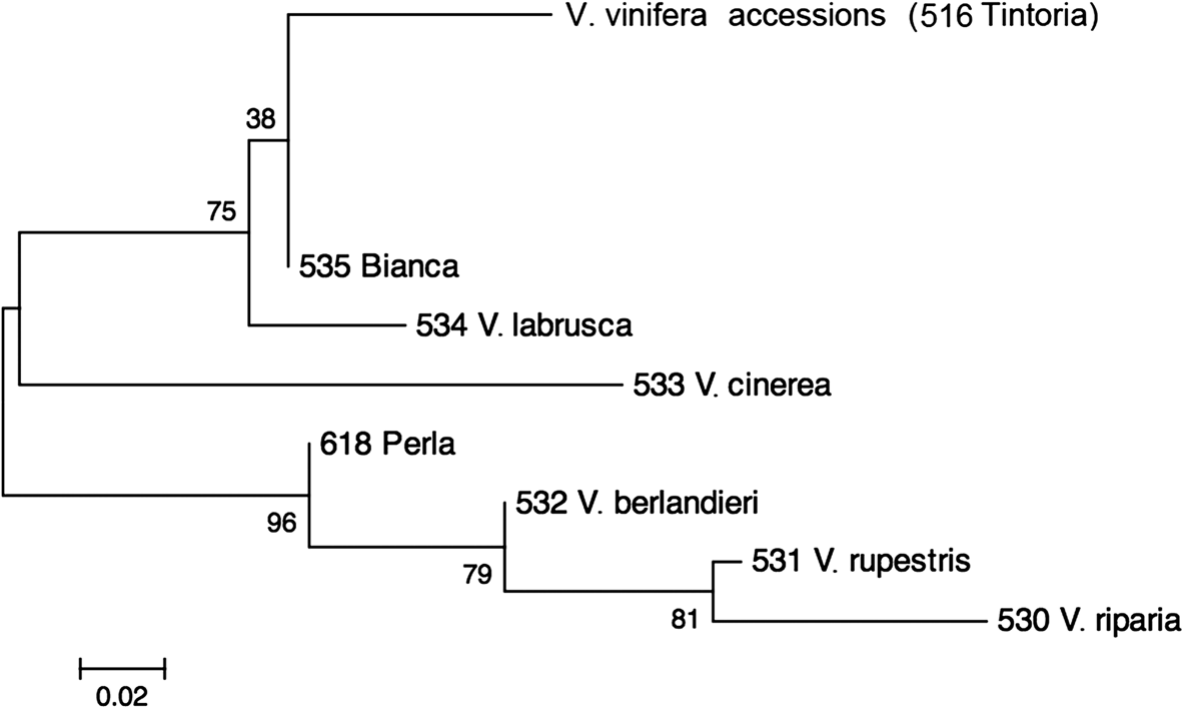
**Part of the Neighbour-Joining tree based on Kimura 2-parameter for 164 grapevine entries belonging to *Vitis *spp. and rooted using as outgroup the accessions from *V. labrusca*, *V. cinerea*, *V. berlandieri*, *V. rupestris *and *V. riparia *species.** In detail, only the branches showing the relationships among *Vitis* spp. and the interspecific hybrids are indicated (see Additional file 3 for the complete Neighbour-Joining tree). The branch labelled with *V. vinifera* accessions indicates the whole accessions of *V. vinifera* and includes also the putative hybrid 516_Tintoria that resulted to be indistinguishable from other cultivated grapevines. The numbers above the nodes represent the bootstrap support after 1,000 replicates.

The NJ tree constructed on the basis of the sequence polymorphisms of all five target barcodes allowed splitting of the accessions of the species *V. labrusca*, *V. cinerea*, *V. berlandieri*, *V. rupestris* and *V. riparia* into distinct main branches with bootstrap values higher than 75% (Figure 4), whereas the branching nodes of *V. vinifera* cultivars were weakly supported (see Additional file 3). Because of the lack of resolution of the NJ tree for *V. vinifera* accessions, a new approach based on genotype reconstruction was tested using the characteristic attributes by exploring the information content of single SNPs.

Owing to the large number of polymorphic sites, it was possible to define a distinct genotype for unambiguously recognizing each one of the five species of *Vitis*. Considering each single gene individually, excluding the non-*V. vinifera* accessions that belong to a specific genotype, the number of genotypes for grapevine cultivars and interspecific hybrids were equal to 18, 28, 11, 19 and 92 for GAI, ID04, IIC08, ATP and UFGT, respectively (see Table 2). When the whole combined sequence was analyzed, each *V. vinifera* cultivar and hybrid could be discriminated and corresponded to distinct genotypes. The most informative marker gene was UFGT, which alone was able to reconstruct 92 genotypes with a number of accessions for each one ranging from 1 to 15 (see Table 2).

Because the accessions used in this study are cultivars under strict selection that do not represent a random sample of grapevine populations following Hardy-Weinberg equilibrium and because a single clone was present for most of the cultivars, all of the variable sites were considered regardless of the restrictive definition that considers a variant a SNP only if the frequency of the most common nucleotide is less than 0.95. In six situations, when multiple individual clones were collected for a given cultivar, we never experienced intracultivar variability and the CAs were shared among all of the representative clones of the cultivar. This situation was true for the cultivars Sultanina, Carmenere, Malbech, Merlot, Pinot Noir and Sagrantino, each of which contained two or three samples that shared the same DNA polymorphisms.

Based on the full combined sequence, frequent compound CAs were detected, allowing for the definition of 116 different genotypes, excluding the five non-*V. vinifera* species (see Additional file 4). Two of them showed peculiar polymorphisms more closely resembling the non-*V. vinifera* species because of the sharing of several highly heterozygous positions. These cultivars were ascribable to Bianca, a recognized interspecific hybrid, and to Perla, initially classified as the certified *V. vinifera* accession Perla of Csaba. For the latter accession, our results would suggest a different origin, more compatible with the Perla of Zala cultivar, an interspecific hybrid between Eger 2, deriving from the French cultivar Villard Blanc introgressed with several *Vitis* species, and Perla of Csaba. Regarding the other *V. vinifera* accessions, the DNA genotyping was also able to distinguish two close cultivars within the Prosecco group: both the GAI and UFGT genes were able to discriminate between Prosecco Lungo and Prosecco Balbi, which originated as two different clones of Prosecco.

Only a few genotypes corresponded to more than one modern cultivar, with a maximum of four cultivars per genotype. It is worth noting that the more numerous genotypes generally grouped either several accessions of the same cultivars (*e.g.*, Merlot, Carmenere and Sultanina) or different strictly related cultivars (*e.g.*, Pinot, Regina or Moscato family). In detail, in the case of Regina the genotype was shared by closely related cultivars, as Razaki, a Regina from Greece and the two Italian cultivars of Regina. Similar results were obtained in the case of the Pinot family, where the two accessions of Pinot Noir (570 and 556), Pinot Blanc and Pinot Gris showed the same CA pattern, for the group of Moscato, which included Moscato Bianco and Moscato Giallo, and for the group of Cannonao. In only one case it was difficult to find a correlation among the cultivars sharing the same pattern of CA: the two cultivars Fiano and Petit Verdot do not share the ancestors, the geographic origin (the first an Italian local cultivar from the Campania region, and the second a French cultivar spread throughout the Veneto and Lazio regions) or the berry colour (Fiano is white, and Petit Verdot is black).

#### Testing local varieties

Once diagnostic genotypes were established based on international references, we tested their utility on some local varieties as case studies to clarify certain genetic relationships among cultivars and eventually resolving situations of synonymy and homonymy. A total of 12 local cultivars grown in the Veneto area generated a specific CAs profile, such as the hybrid Tintoria, Schiavetta Doretta and Marzemina Nera Bastarda, whereas the remaining 11 accessions shared the nucleotide composition of the genotype with at least another cultivar, local or international. For example, we were able to confirm the origin of some local accessions by comparing their genotypes with those present in the developed reference system. The accessions 552 and 558 were found to correspond to two certified cultivars, Raboso Piave and Raboso Veronese, respectively, and they could be distinguished from each other by belonging to two different genotypes. Using the combined SNPs, the local genotypes labeled as Raboso Piave, 522 and 523, clustered together with the reference standard 552_Raboso Piave, thus confirming the SSR results of Salmaso et al. [45], and the local 524_Raboso Veronese was identical to 558_Raboso Veronese except for one nucleotide site. In addition, the Friularo cultivar was collected, and five different clones from as many farmers were sampled. By the CAs reconstruction, four out the five clones grouped together in the same genotype including 552_Raboso Piave, while the 521_Friularo7 grouped with 558_Raboso Veronese. An additional finding obtained by nuclear gene sequencing was the genetic equivalency between the cultivars Marzemina Nera and Marzemina Cenerenta, Corbinona and Corbinella, and Cabernet Lispida and Carmenere, which share the same SNP-based genotypes.

### Discussion

#### Developing a reference system by means of DNA barcoding

The use of DNA barcoding to test the genetic distinctiveness of grapevine cultivars, and crop varieties in general, is a recent application of the technique that is under study. In fact, DNA barcoding was initially proposed as a diagnostic tool to determine the species identity of unknown organisms. In this paper, its ability to distinguish modern varieties within *V. vinifera* species was tested, an application that could reveal of great utility due to the agronomic importance of the crop. An additional feature of the DNA barcoding was tested such as its capacity to characterize different biotypes within the same cultivar. The concept of biotype employed in the study refers to a genotype that differentiated genetically from the original cultivar through occurrence of gemmary mutations, epigenetic effects or their combination, determining the acquisition of a new and well-recognizable morphological or physiological trait.

The analysis of 164 grapevine accessions was performed by the character-state method because the application of the conventional phenetic approach showed to be unsuitable for an intraspecies assay, as proven by the low genetic distance within *V. vinifera* calculated using the K2P parameter. Distinguishing genetic entities below the species level requires a more sensitive approach that is able to conserve all sequence information without converting them into genetic distances. Furthermore, the balance sought for DNA markers is such that within-species genetic diversity is minimized, but in this study it was of principal importance. Thus, we combined the sequencing of chloroplast barcode regions and nuclear single-copy genes with the more robust SNP-based DNA genotyping method to better define the boundaries among agronomically important cultivars.

The first attempts aimed at discovering genetic diversity among cultivars were conducted on the haploid chloroplast genome, but it proved to be not sufficiently variable to allow the reconstruction of distinct haplotypes for individual varieties within the species. The alternative approach was based on the sequencing of single-copy genes from the nuclear genome, which shows synonymous substitution rates generally greater than those found for plastid and mitochondrial genes [46]. The analysis of the nuclear genome became very common in the last few decades due to DNA recombination and biparental inheritance pattern, which allows shifting from the gene trees to a multi-locus study of population history [47]. In addition, nuclear DNA offers the advantage of resolving problems associated with the horizontal acquisition of organelles through hybridization events or with introgression patterns that can be detected only using biparental markers [48]. Importantly, this approach needs a preliminary selection of single-copy genes to be used as DNA markers.

An intrinsic problem of using nuclear sequences is the difficulty of interpreting the frequent occurrence of additive cases that can often lead to misinterpretation of the results. Because we were working with *V. vinifera* species, a highly heterozygous diploid species, frequent cases of intragenomic variation were detected because of the presence of more than one allele variant for a particular locus. Generally, with the presence of heterozygous sites, it would be necessary to separate the allele variants and to define the nucleotide associations for the polymorphic sites. In the specific case of *V. vinifera* we combined all SNPs of both alleles for each locus in a single sequence and therefore we employed the concept of genotype, in place of haplotype. In addition, since *V. vinifera* species is maintained by vegetative propagation and thus the genetic recombination is negligible, the genomic DNA patrimony is fixed, allowing the definition of a specific genotype for each grapevine cultivar.

Considering all the certified samples, 121 genotypes were discovered: five were able to distinguish the wild *Vitis* species, one was specific for the hybrid Bianca, as many as 109 were cultivar-specific and the remaining six genotypes were ascribable to several cultivars at the same time. Regarding the possibility of using SNP genotyping to distinguish among closely related genotypes, such as the Pinot, Moscato, Regina and Cannonao groups, this ability remains challenging. The Pinot family, for example, includes the original cultivar, Pinot Noir, which has black berries, and the two cultivars, Pinot Gris and Pinot Blanc, that are thought to be chimeras, mutant clones derived from Pinot Noir after the occurrence of a mutation for berry colour in one cell layer of the berry for Pinot Gris (red-grey berry) and in both of the cell layers for Pinot Blanc (white berry). These kinds of somatic mutations are very common in grapevine and contribute to the high incidence of genetic variability. Because of the origin of this mutation, probably the only way to resolve the genetic recognition of these three cultivars would be the individuation of a marker map for the gene controlling berry colour and the mutation responsible for the colour change. Even if UFGT belongs to the biosynthetic pathway of anthocyanins, a retrotransposon-induced mutation in the transcription factor-coding gene *VvmybA1* is the molecular basis of the white coloration, as demonstrated by Pereira et al. [49] and Furiya et al. [50]. Thus, there are important limits to the resolution of homonymy situations we may obtain with genetic markers alone. Regarding the other possible cause for the multi-cultivar genotypes, the occurrence of these groups could further corroborate some theories suggesting cases of synonymy or parent-offspring relationships. For example, the two Italian cultivars Nero d’Avola and Calabrese are known to be synonymous, and their genomic composition matched, even though the complete sequence of the Calabrese accession was not available. Regarding possible offspring relationships, the identical SNP compositions of Alphonse Lavallèe and Palieri, except for one position, could be explained by the fact that Palieri is the offspring of Alphonse Lavallèe × Red Malaga (a cultivar not present in this study). In addition, Raboso Veronese is the offspring of Raboso Piave × Marzemina Bianca, and the nucleotide composition of Raboso Veronese is in agreement with its origin (see Additional file 1). Finally, the genomic composition of the two accessions Bianca and Perla could be explained by their phylogenetic origin as interspecific hybrids with other non-*V. vinifera* accessions. Despite these considerations, it is very difficult to reconstruct the pedigree and develop hypotheses about offspring relationships. In fact, sequencing of nuclear markers proved to be a valid genomic tool to distinguish species and, in large extension, cultivars. Nevertheless, the exploitation of this technique to infer offspring relationships seems risky because of the limited number of available SNPs.

Using STRUCTURE software, it was possible to identify four putative subpopulations and to probabilistically assign individuals to the corresponding clusters on the basis of their genotypes. Similarly to what reported by Emanuelli et al. [14], a stratification structure was observed being the primary division between accessions of *V. vinifera* and *V*. non-*vinifera*, followed by the distinction of intra-specific clusters within cultivated grapevine accessions. Nevertheless, the analysis of overall data revealed that there is no relationship between the cultivation area of the cultivars and the genomic composition. Only the wild *Vitis* species were clustered in a specific sub-group, together with the Perla accession, whereas all the cultivars were assigned almost in equal proportion of membership to the three *V. vinifera* sub-groups identified revealing an admixed origin. Regarding Perla, although the analysis was conducted considering this accession a *V. vinifera* cultivar, our results highlighted a different origin more compatible with an interspecific pedigree. In fact two different accessions, generally called Perla, are commercially available: Perla of Csaba, a *V. vinifera* variety, and Perla of Zala, an interspecific hybrid between Perla of Csaba and Eger2 cultivar deriving from Villard Blanc, a France hybrid whose genetic patrimony derives from several *V.* non-*vinifera*. The genomic composition of this variety supports the hypothesis that our material belongs to the Perla of Zala cultivar. Similarly to Perla, also Bianca cultivar showed a considerable contribution of the *V*. non-*vinifera* species, even if with less extension, to its nucleotide composition supporting in this way its interspecific origin. In fact, Bianca is a hybrid deriving from the backcross of the French *V. vinifera* cultivar Villard Blanc with its ancestors, which include germplasm of *V. aestivalis*, *V. berlandieri*, *V. cinerea*, *V. lincecumii* and *V. rupestris*, accessions used to introduce the resistance genes of the North America grapes [51].

#### Genotyping single-copy nuclear genes for the molecular characterization of local germplasm

Once specific genotypes were identified among the international cultivars used as standard references, an additional sampling of ancient local varieties typical of northeastern Italy was performed to include them in the analysis. Characterizing this local germplasm, which represents a valuable genetic resource for the region, would be the first step of a conservation policy aimed at the preservation and valorization of old native cultivars. The identification and description of this local patrimony represents not only a valuable resource for the territory, because some local cultivars still constitute the basis of famous regional wines such as Gruaja or Marzemina, but also a potential source of genetic variability exploitable for genetic improvement programs (breeding schemes assisted by molecular markers), providing the information required for the correlation of molecular markers with phenotypic distinctive traits of grapevine cultivars. The employment of our varietal germplasm collection can be considered an explorative assay to test the effectiveness of sequencing nuclear genes to examine the genetic identity of samples, eventually resolving cases of synonymy and homonymy, and to compare the results emerging from nuclear genes with those previously obtained by using nuclear and chloroplast SSR markers [38,45].

Among the 23 local cultivars employed in this study, five are registered in the Italian Catalogue of Cultivated Varieties: Pignola, Marzemina Bianca, Marzemina Nera, Raboso Piave and Raboso Veronese. The other cultivars were developed in the Veneto regional area, where they are best adapted and still cultivated, thus belonging to a genetic patrimony that needs to be characterized, preserved and valorized. By means of SNP markers, it was possible to reconstruct specific genotypes for the Tintoria hybrid and for 11 *V. vinifera* local cultivars, of which three were registered in the Italian Catalogue and the other eight were not. For those genotypes that clustered many cultivars at the same time, cases of synonymy can be hypothesized. For instance, the cultivars Corbinona and Corbinella proved to share the same nuclear composition, confirming former results obtained by nuclear and chloroplast SSR markers that demonstrated synonymy, except for one allele, between these two cultivars [45]. A similar finding was observed for the cultivars Marzemina Nera and Marzemina Cenerenta, which are characterized by synonymy on the basis of previous SSR studies [45]. In the Raboso group, the local non-certified genotypes clustered with the proper international reference standards, confirming their genetic identity with these cultivars. A particular case is the cultivar Friularo, which is not registered in the Italian Catalogue and is recognized as a biotype of Raboso Piave adapted to the Euganean regional area. In fact, these two cultivars are genetically indistinguishable using both SSR genotyping [45] and sequencing techniques. A probable labeling mistake was found for the 521_Friularo7 cultivar, which instead of clustering with the other Friularo and Raboso Piave cultivars, grouped with the two Raboso Veronese cultivars. The local cultivar Tintoria was considered to be an interspecific hybrid with non-*V. vinifera* accessions. In fact, this cultivar on the basis of chloroplast SSR markers showed tight relationships with American grapevine species [45] and the nuclear DNA sequencing further supports this hypothesis. Finally, the ancient cultivar Gruaja, whose cultivation has almost disappeared and narrowed to a small area of the Vicenza province, was characterized by a high incidence of mutations. Preserving ancient cultivars is fundamental for genetic improvement programs because, due to the fact that these cultivars likely have been accumulating and fixing more mutations than young cultivars, the high incidence of mutations can be the starting point for the origin of new alleles. In addition, the chimeric situation can represent an interesting source of clonal variability and its recovery can contribute to the generation of new agronomically useful phenotypes.

Concluding, the high number of genotypes obtained so far demonstrates that the nuclear genome is variable enough to function as a source of diagnostic markers for characterization issues, allowing the genetic authentication of 130 *V. vinifera* genotypes of which 115 belonging to international cultivars and 15 to local varieties. DNA sequencing, based on nuclear markers, proved to be very effective in distinguishing grapevine cultivars, except in the case of closely related cultivars such as within the Pinot family, or to reflect the phylogeographic history of the biotypes, as in the case of the Regina or Cannonao groups. The large portion of the UFGT gene assayed in this study proved to be the most polymorphic and discriminant marker and thus it deserves deep attention because our data suggest that the coding region of this single-copy nuclear gene alone is sufficiently informative for a single-locus sequence genotyping analysis applied at the intraspecies level for assessing grapevine cultivar identity and ancestry.

## Availability and requirements

### Webpages

BLAST: Basic Local Alignment Search Tool; Available from: http://www.ncbi.nlm.nih.gov/

*Vitis* International Variety Catalogue (VIVC); Available from: http://www.vivc.de.

## Competing interests

Authors declare to have any competing interests in this manuscript.

Financial competing interests: In the past five years, authors did not receive reimbursements, fees, funding, or salary from an organization that may in any way gain or lose financially from the publication of this manuscript, neither we will not receive any in the future. Any organization is financing our manuscript. We do not hold any stocks or shares in an organization that may in any way gain or lose financially from the publication of this manuscript, neither we will hold any in the future. We do not hold and we are not applying for any patents relating to the content of the manuscript. We have not received reimbursements, fees, funding, or salary from an organization that holds or has applied for patents relating to the content of the manuscript. None of the authors have any other financial competing interests.

Non-financial competing interests: Authors do not have any non-financial competing interests (political, personal, religious, ideological, academic, intellectual, commercial or any other) to declare in relation to this manuscript.

## Authors’ contributions

SN performed all the experiments, analyzed the sequence data and wrote the first draft of the manuscript. GB conceived the study, designed the experiments, and collaborated to data analysis, and preparation and revision of the manuscript. JK and DE supervised the DNA barcoding work and revised the manuscript. ML devised the study, collaborated to the selection of plant materials and to the preparation of the manuscript. All authors read and approved the final manuscript.

## Supplementary Material

Additional file 1 List of 164 grapevine entries with the common name of the cultivars along with the origin area, source of genetic germplasm and destination of the berry. Click here for file

Additional file 2 Proportions of membership to the four inferred clusters of each individual grapevine accession pre-attributed to different subgroups according to their geographical origin. Click here for file

Additional file 3 **Neighbour-Joining full tree based on Kimura 2-parameter including all 159 grapevine entries of *Vitis vinifera*, rooted using as outgroup the accessions from *V. labrusca*, *V. cinerea*, *V. berlandieri*, *V. rupestris* and *V. riparia* species.** Click here for file

Additional file 4 **Single nucleotide polymorphisms (107 CAs) identified in the five target nuclear regions with information on the composition of genotypes found across all grapevine (*Vitis* spp.) entries.** Click here for file
