# Supplementary figures and images for: The coding region of the UFGT gene is a source of diagnostic SNP markers that allow single-locus DNA genotyping for the assessment of cultivar identity and ancestry in grapevine (Vitis vinifera L.)
Source: BMC Res Notes. 2013 Dec 3;6:502. doi: 10.1186/1756-0500-6-502 (PMC4222114; doi:10.1186/1756-0500-6-502)

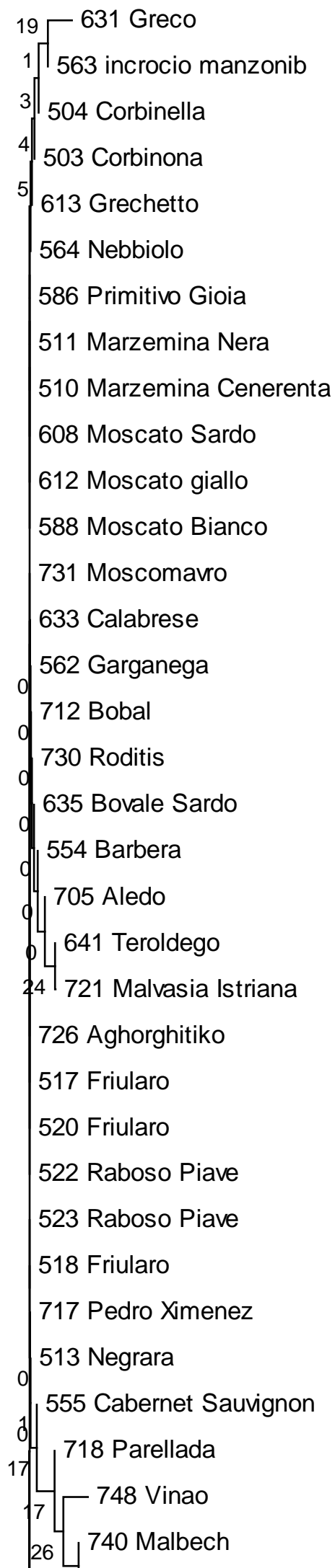

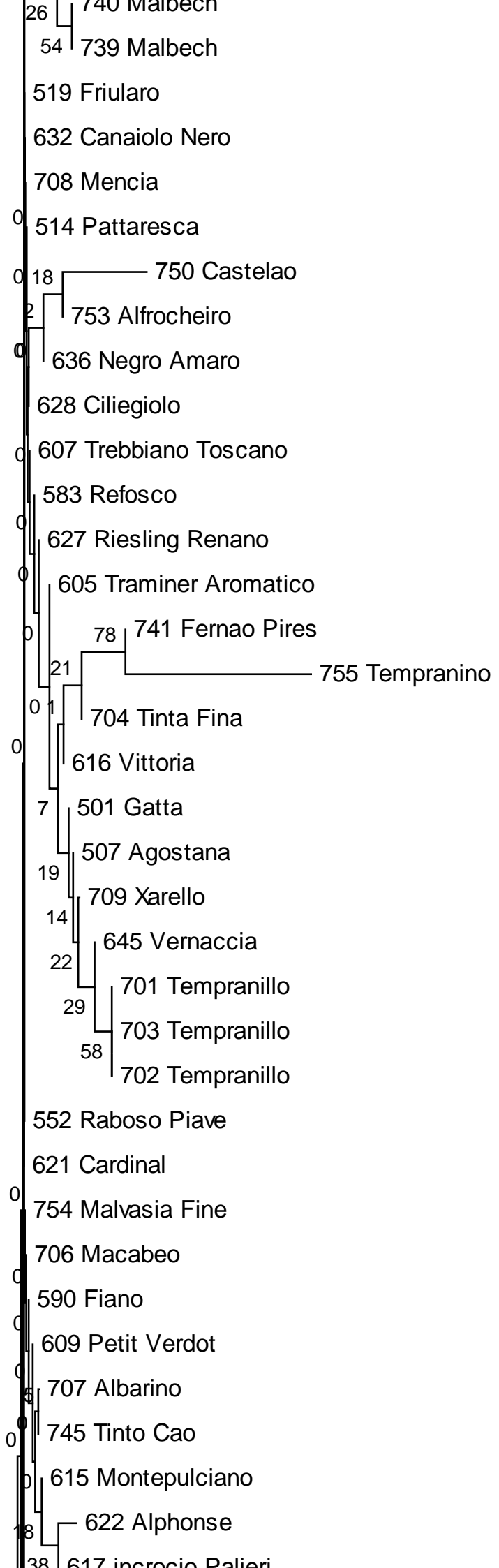

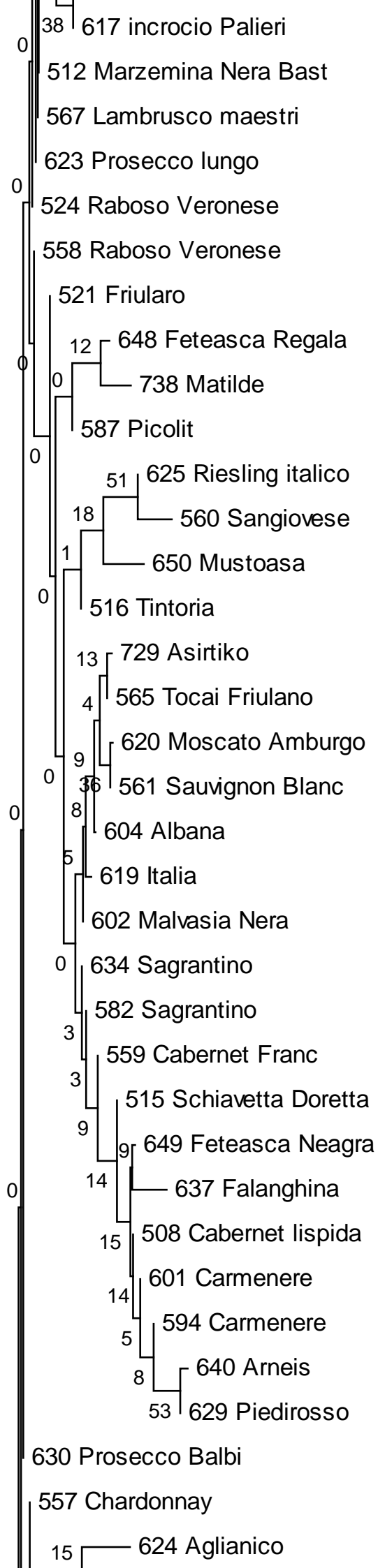

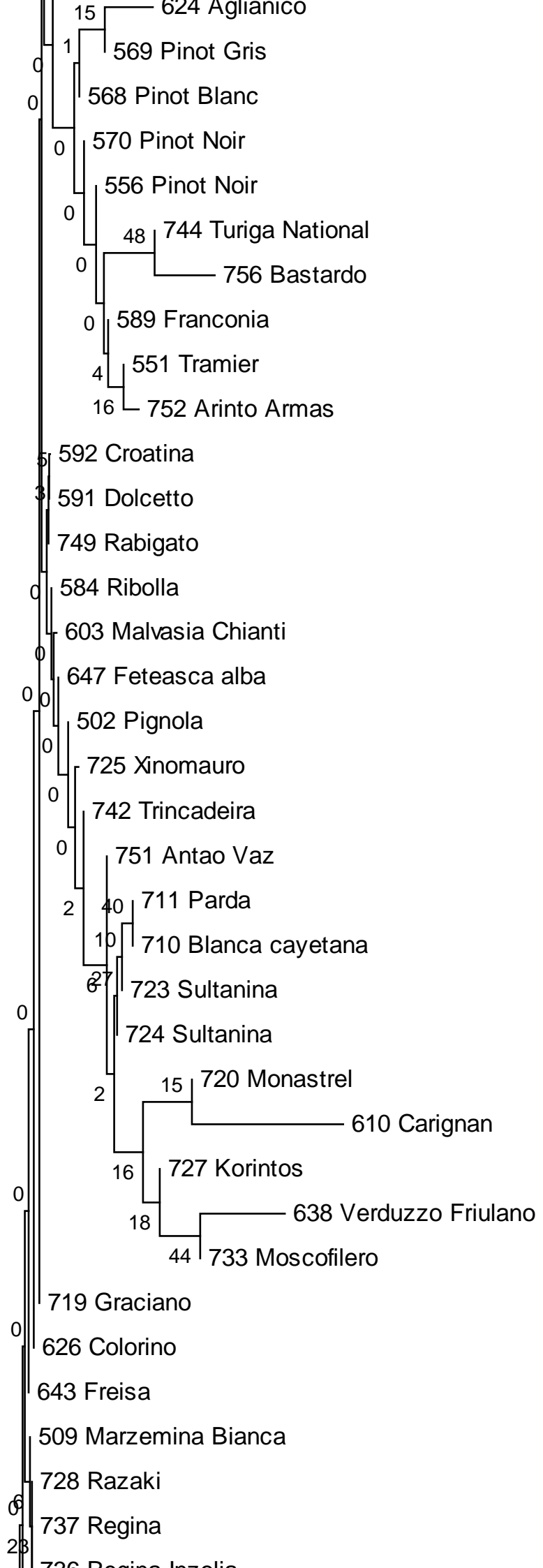

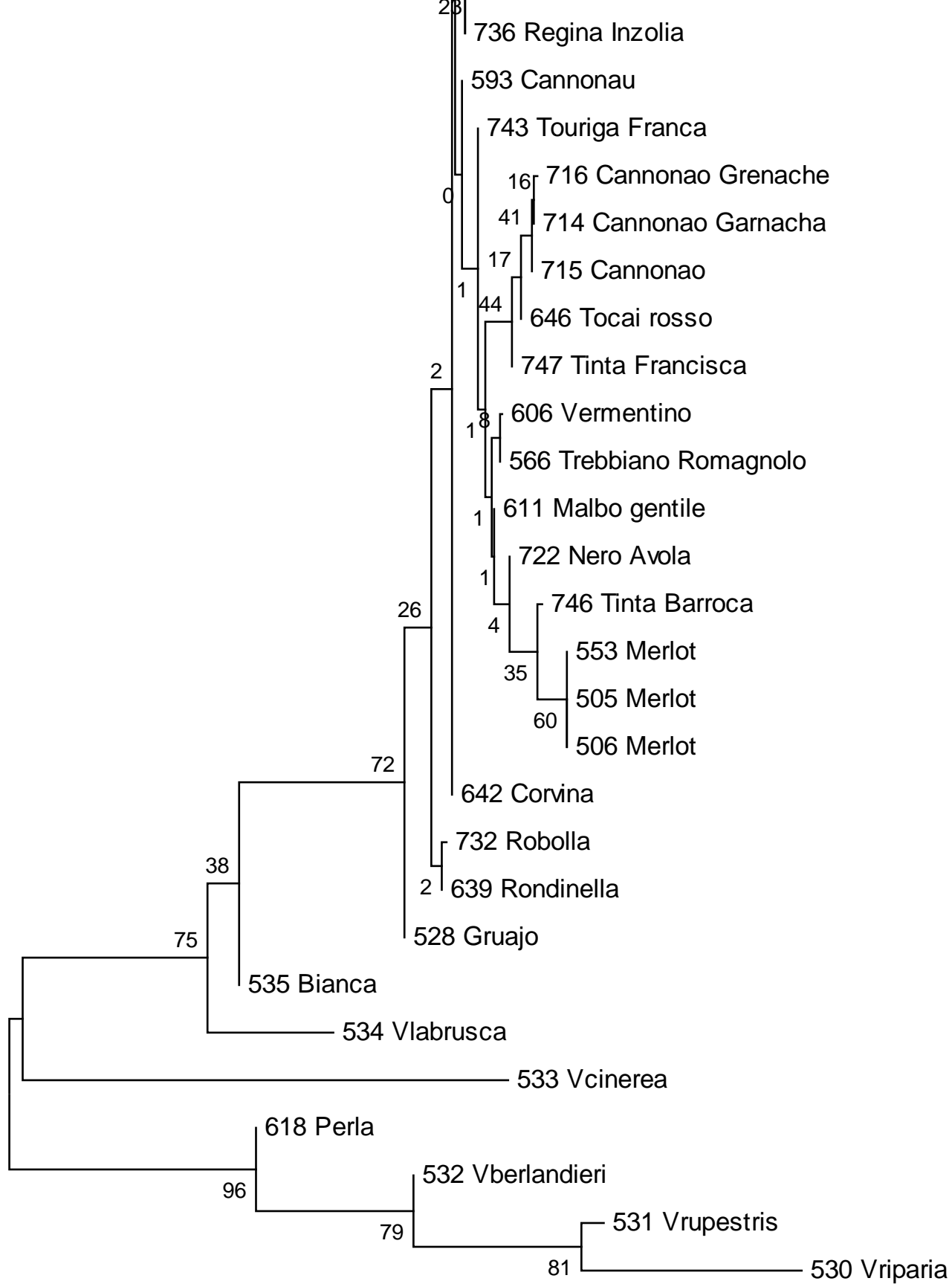

0.02

Supplement: Additional file 3 — Neighbour-Joining full tree based on Kimura 2-parameter including all 159 grapevine entries of Vitis vinifera , rooted using as outgroup the accessions from V. labrusca , V. cinerea , V. berlandieri , V. rupestris and V. riparia species. [file 1756-0500-6-502-S3.pdf]
